# Ligation Motifs in Zinc-Bound Sulfonamide Drugs Assayed by IR Ion Spectroscopy

**DOI:** 10.3390/molecules27103144

**Published:** 2022-05-14

**Authors:** Davide Corinti, Barbara Chiavarino, Philippe Maitre, Maria Elisa Crestoni, Simonetta Fornarini

**Affiliations:** 1 Dipartimento di Chimica e Tecnologie del Farmaco, Sapienza Università di Roma, P.le A. Moro 5, I-00185 Roma, Italy; barbara.chiavarino@uniroma1.it (B.C.); mariaelisa.crestoni@uniroma1.it (M.E.C.); 2 Institut de Chimie Physique, UMR8000, CNRS, Université Paris-Saclay, 91405 Orsay, France; philippe.maitre@universite-paris-saclay.fr

**Keywords:** sulfadiazine, sulfathiazole, zinc coordination, sulfonamide antibiotics, structure determination, metal complexes, FTICR mass spectrometry, IRMPD spectroscopy, DFT calculations

## Abstract

The sulfonamide–zinc ion interaction, performing a key role in various biological contexts, is the focus of the present study, with the aim of elucidating ligation motifs in zinc complexes of sulfa drugs, namely sulfadiazine (SDZ) and sulfathiazole (STZ), in a perturbation-free environment. To this end, an approach is exploited based on mass spectrometry coupled with infrared multiple photon dissociation (IRMPD) spectroscopy backed by quantum chemical calculations. IR spectra of Zn(H_2_O+SDZ−H)^+^ and Zn(H_2_O+STZ−H)^+^ ions are consistent with a three-coordinate zinc complex, where ZnOH^+^ binds to the uncharged sulfonamide via N(heterocycle) and O(sulfonyl) donor atoms. Alternative prototropic isomers Zn(OH_2_)(SDZ−H)^+^ and Zn(OH_2_)(STZ−H)^+^ lie 63 and 26 kJ mol^−1^ higher in free energy, respectively, relative to the ground state Zn(OH)(SDZ)^+^ and Zn(OH)(STZ)^+^ species and do not contribute to any significant extent in the sampled population.

## 1. Introduction

Since their discovery in 1935, sulfonamide drugs, derivatives of 4-aminobenzenesulfonamide (H_2_N-C_6_H_4_-SO_2_NH_2_), have been used extensively as wide-spectrum antibiotics for the treatment of human and animal bacterial infections [1,2,3,4]. Their bacteriostatic action is exerted by inhibiting the use of 4-aminobenzoic acid, which is essential for the synthesis of folic acid, a fundamental developmental factor in the metabolism of microbes. Sulfonamides are currently widely used as veterinary antibiotics, and their release in the environment raises serious ecotoxicity concerns [5,6]. Transition metal complexes of sulfa drugs have shown enhanced antibacterial, antifungal and antiglaucoma action [7,8]. Coordination to zinc is an underlying motif in the pharmacological activity of sulfonamides. For example, zinc sulfonamides have been successfully used for treatment of microbial and fungal infections in burn wounds and found to promote wound healing [9,10]. Notably, zinc resides at the active site of carbonic anhydrase (CA) enzymes, which catalyze the reversible hydration of carbon dioxide to hydrogen carbonate ions. Many therapeutic applications rely on CA inhibition by ligand coordination to the active site zinc. The majority of inhibitors is based on zinc binding via a deprotonated primary sulfonamide function (RSO_2_NH^−^) [11,12,13]. The interaction of sulfonamide inhibitors and human CA I has been addressed recently using native mass spectrometry, which allows for preservation of non-covalent interactions during the transition of the protein–ligand complex from solution to the gas phase. In this way, stoichiometric information about the composition of the complex was obtained, and the binding of different inhibitors was clarified [14].

In a recent report, the protonation site in representative sulfonamide drugs was thoroughly assayed by infrared multiple photon dissociation (IRMPD) spectroscopy [15]. Interestingly, the favored protonation site in the parent molecule of the sulfa drug family, namely sulfanilamide (H_2_N-C_6_H_4_-SO_2_NH_2_), is highly sensitive to the environment. However, sampling of the gaseous ion has unambiguously revealed that protonation occurs on the amido nitrogen, yielding the most stable isomer in the gas phase. This finding suggests that a proton shift occurs on protonated sulfanilamide formed in solution, where the added proton is known to reside on the aniline NH_2_ group upon transfer from solution to the gas phase during the electrospray ionization (ESI) process.

**Scheme 1:**
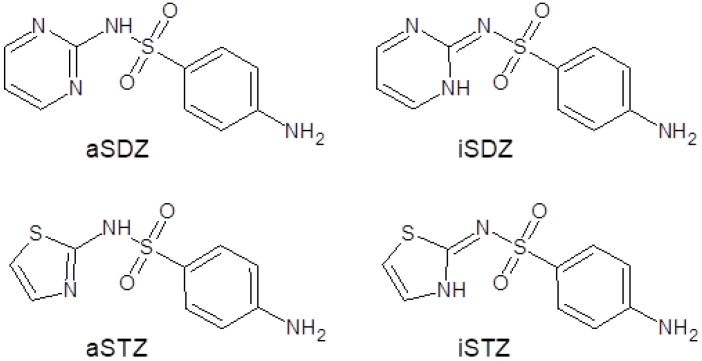
Amido (aSDZ and aSTZ) and imido (iSDZ and iSTZ) tautomers of SDZ and STZ.

Sulfathiazole (STZ) and sulfadiazine (SDZ), the sulfa drugs that are specifically addressed in this contribution, belong to the family of clinically used sulfonamides for the treatment of infectious diseases [1]. Their structure, comprising two tautomeric (amido and imido) forms [16], is depicted in Scheme 1.

In view of the mutual relationship between zinc ion and sulfonamide (SA) drugs and the relevance of their bonding in the ensuing bioinorganic chemistry, the structure of isolated, charged Zn/SA complexes is examined using IRMPD spectroscopy and quantum chemical calculations. IRMPD spectroscopy provides vibrational spectra of gaseous ions based on the dissociation of the sampled species when it is activated by the absorption of multiple IR photons in resonance with an active vibrational mode. Only when the resonance condition is met do the sampled ions acquire internal energy in a stepwise fashion, consisting of multiple IR photon absorption events, each of which is followed by intramolecular vibrational relaxation (IVR). IVR allows for restoration of resonance with the IR active mode, which permits the ion to augment its internal energy until a fragmentation threshold is reached. Product ions are revealed by mass spectrometry, and their occurrence confirms the matching between an active vibrational mode of the precursor ion and the radiation frequency [17,18,19,20,21]. The photofragmentation process to which ions trapped in the cell of a mass spectrometer are subjected, relies on the high fluence of a laser source, such as the free-electron laser at the Centre Laser Infrarouge d’Orsay (CLIO), where the present data were obtained. IRMPD spectroscopy has been demonstrated to afford a valuable characterization of biologically active molecules and molecular complexes since its expansion as a structural diagnostic tool of mass-selected charged species [17,18,19,20,21]. In a considerable number of studies, including contributions from these authors, it has been applied to identify binding motifs and preferred geometries of transition metal complexes coordinating biomolecular ligands and targets [22,23,24,25,26,27,28,29,30,31,32]. Zinc complexes of biomolecules have also been assayed [33,34,35,36,37,38]. In particular, the structural characteristics of Zn^2+^ cationized amino acids were evaluated in a series of studies [33]. A tridentate structure is observed from zinc complexation of L-methionine, whereas an additional chlorido or acetonitrile ligand yields a four-coordinate zinc complex. In the zinc-bound histidine (His) dimer, Zn(His−H)(His)^+^, the deprotonated His chelates the metal by the amino nitrogen, the aza nitrogen of the imidazole side chain and the deprotonated carboxyl oxygen, and the intact His ligand coordinates the metal via the two carboxylate oxygens belonging to a zwitterionic structure [34]. A tetrahedral-type coordination environment has been reported for the phenylalanine (Phe) complex Zn(Phe−H)(Phe)^+^, where Zn^2+^ binds to the N and O atoms of both ligands, conforming to the strong preference of zinc for tetrahedral binding sites in proteins [35]. A four-coordinate zinc complex is also evidenced in the IRMPD spectrum of the zinc-bound uracil (Ura) dimer Zn(Ura−H)(Ura)^+^, where uracil deprotonation occurs at N3 [36]. The formal Zn(Ura−H)(H_2_O)^+^ complex displays an IRMPD spectrum that is accounted for by the lower-lying Zn(Ura)(OH)^+^ species [36]. IRMPD spectroscopy has helped to elucidate the structures of the proline (Pro) complexes Zn (Pro−H)^+^ and Zn(Pro−H)(H_2_O)^+^ in the gas phase, pointing to the migration a hydrogen atom, forming a Zn-H bond [37].

In view of the context described about and the interest attached to zinc complexes of sulfonamide drugs, the positively charged complexes formally derived from Zn^2+^ bound to H_2_O and SDZ or STZ, where either the aquo or the sulfa drug ligand is deprotonated, are assayed by FT-ICR mass spectrometry coupled with IRMPD spectroscopy and quantum chemical calculations. Their composition is represented by the formulas Zn(H_2_O+SDZ−H)^+^ and Zn(H_2_O+STZ−H)^+^ for the SDZ and STZ complexes, respectively, where the ligand being deprotonated is yet undefined. Structural and spectroscopic features of deprotonated SDZ were also been inspected and are reported for comparison purposes.

## 2. Results and Discussion

### 2.1. Mass Spectrometry and Photofragmentation Patterns

IRMPD spectroscopy, considered an “action” spectroscopy, relies on a fragmentation event that is activated by the absorption of multiple IR photons when their frequencies match with an active vibrational mode of the sampled ion [39,40]. An outline of the photofragmentation pattern displayed by the ions of interest is briefly described herein. The electrospray ionization (ESI) mass spectrum of SDZ in negative ion mode presents a distinct signal at *m*/*z* 249 corresponding to the monoisotopic peak of deprotonated SDZ, namely (^12^C_10_H_9_^14^N_4_^16^O_2_^32^S)^−^. Isolation and irradiation by IR photons in resonance with an active vibrational mode yields a single significant fragment at *m/z* 185, as shown in Figure S1 in the Supplementary Material. Photofragmentation involves loss of SO_2_, a process commonly observed in the mass spectra of sulfonamides and their derivatives under electron ionization [41], as well as in the negative ESI mass spectrometry of sulfonamides, for which a fragmentation mechanism has been proposed [42].

Ions corresponding to Zn(H_2_O+SDZ−H)^+^ present a monoisotopic peak of (^12^C_10_H_11_^14^N_4_^16^O_3_^32^S^64^Zn)^+^ composition at *m*/*z* 331 and are characterized by an isotopic pattern congruent with zinc isotope distribution (^64^Zn(48.6%), ^66^Zn (27.9%), ^67^Zn (4.1%), ^68^Zn (18.8%) and ^70^Zn (0.6%)). When Zn(H_2_O+SDZ−H)^+^ ions are subjected to IRMPD, the observed photofragmentation pattern comprises product ions retaining the zinc atom, as evidenced by the characteristic isotope distribution (Figure S2). The predominant product at *m*/*z* 251 is due to the loss of 80 Da, which is thought to involve either a pyrimidine molecule (C_4_H_4_N_2_) or SO_3_. However, the accurate mass analysis allowed by FT-ICR mass spectrometry [43] revealed a mass loss from precursor to product ion amounting to 79.965 Da, which is in line with a SO_3_ (79.957 Da) fragment, disproving the C_4_H_4_N_2_ (80.037 Da) alternative. A second major product at *m*/*z* 313 is due to the departure of a water molecule. Only in the presence of extensive photofragmentation are ions at *m*/*z* 295 (by loss of two water molecules from the precursor ion) and *m*/*z* 158 observed. The latter species, formally corresponding to a zinc ion bound to deprotonated aminopyrimidine (C_4_H_4_N_3_Zn)^+^, is related to the ion at *m*/*z* 313 by a 155 Da fragment, which is a typical loss found in the collision-induced dissociation mass spectrum of protonated sulfadiazine and other sulfonamides [44,45].

The monoisotopic peak (^12^C_9_H_10_^14^N_3_^16^O_3_^32^S_2_^64^Zn)^+^ at *m*/*z* 336 pertaining to Zn(H_2_O+STZ−H)^+^ ions, when submitted to photofragmentation, yields product ions at *m*/*z* 318 (loss of water), *m*/*z* 256 (loss of SO_3_, supported by the same arguments explained for the analogous SDZ complex), *m*/*z* 254 (by formal loss of H_2_SO_3_) and *m*/*z* 190 (by formal elimination of ZnO + H_2_SO_2_), as shown in Figure S3. Multiple rearrangement and fragmentation processes are well documented in the tandem mass spectra of protonated sulfonamides [46,47,48].

### 2.2. Structural and Vibrational Features of Deprotonated Sulfadiazine, (SDZ−H)^−^, Ions

**Figure 1:**
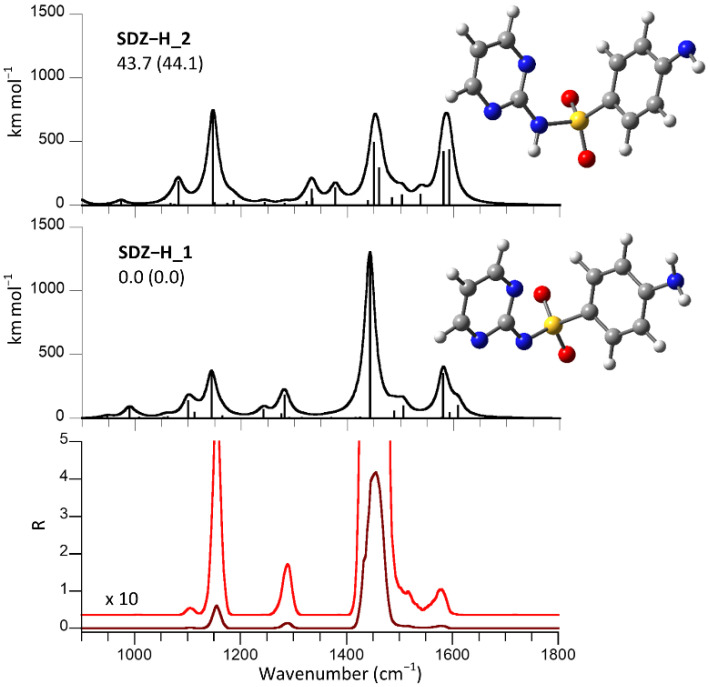
IRMPD spectrum of (SDZ−H)^−^ (bottom panel, dark and pale red profiles) compared with calculated IR spectra of **SDZ−H_1** and **SDZ−H_2**, the optimized structures of which are reported on the right. Relative free energies (enthalpies) at 298 K are reported in kJ mol^−1^. Calculations are at the B3LYP/6-311+G(2df,pd) (S = 6-311+G(3df)) level. Computed harmonic frequencies are scaled by a factor of 0.974, except those regarding S-X stretching modes, which are left unscaled.

Sulfadiazine behaves as a weak acid in water, where deprotonation at the amido group, activated by the powerful electron-withdrawing sulfonyl function, is characterized by a pK_a_ of 6.5 [49]. The IRMPD spectrum recorded on the (SDZ−H)^−^ species shown in the lower panel of Figure 1 is thus expected to pertain to the amido deprotonated anion. To confirm this view, DFT calculations were performed. The optimized structure named **SDZ−H_1** displays a theoretical IR spectrum that accounts well for the experimental features (Figure 1). The geometry of **SDZ−H_1** is reminiscent of the two most stable conformers of neutral sulfadiazine, differing for the orientation of the amino group relative to the phenyl ring [50]. Vibrational frequency analysis allows the observed bands to be assigned, as summarized in Table 1. The most pronounced band at 1456 cm^−1^ is mainly associated with the C(pyr)-NSO_2_ stretch calculated at 1442 cm^−1^. The second major feature at 1156 cm^−1^ is due to the SO_2_ symmetric stretch, for which the calculated harmonic frequency is 1144 cm^−1^. Because the amino group of sulfadiazine may be another source of mobile protons, an amino-deprotonated isomer, **SDZ−H_2**, has was also considered. The IR spectrum for the **SDZ−H_2** optimized structure reveals a pattern that is not compatible with the experiment. Most evident is the presence of a distinct IRMPD feature at 1289 cm^−1^ that is well interpreted by the SO_2_ asymmetric stretch of **SDZ−H_1** expected at 1289 cm^−1^ but that falls in an almost blank region of the IR spectrum of **SDZ−H_2**. For comparison purposes, it is worth noting that the SO_2_ asymmetrical/symmetrical stretches are observed at 1326 cm^−1^ and 1157 cm^−1^ in the IR spectrum of neutral sulfadiazine, respectively [50].

Besides confirming the deprotonation site in sulfadiazine, the fair matching between the experimental IRMPD and the calculated IR spectrum supports the adopted computational approach as appropriate for an adequate description of the assayed set of molecular ions.

### 2.3. Structural and Vibrational Features of Zn(H_2_O+SDZ−H)^+^ Complexes

**Figure 2:**
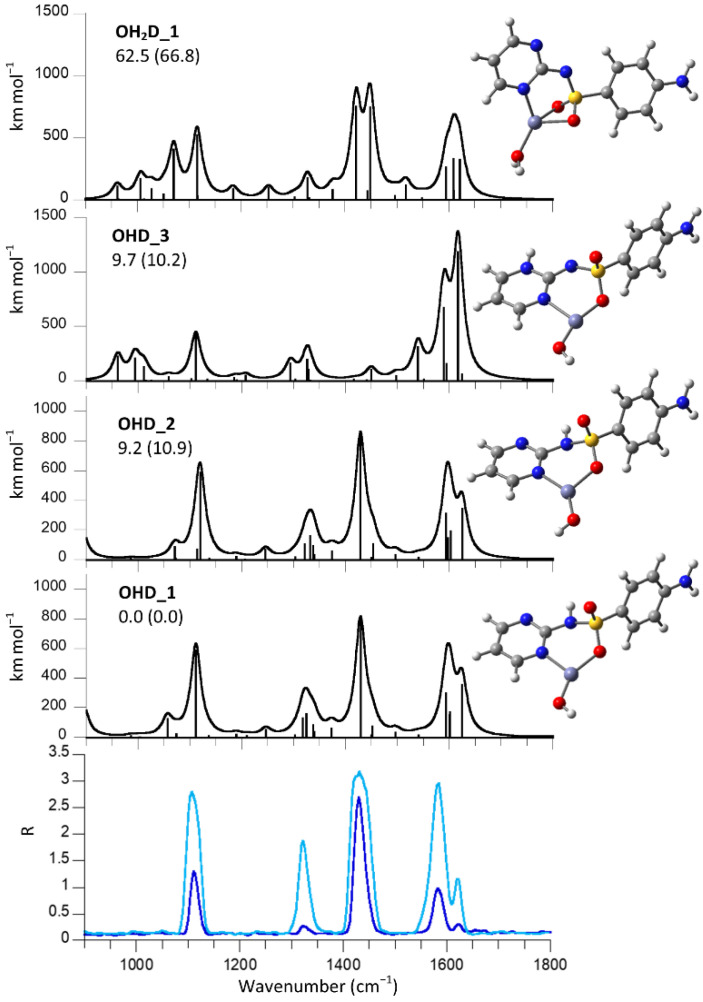
IRMPD spectrum of Zn(H_2_O+SDZ−H)^+^ (bottom panel; dark and pale blue profiles were recorded with and without the use of one attenuator (−3 dB), respectively) compared with calculated IR spectra of selected isomers, the optimized structures of which are reported on the right. Relative free energies (enthalpies) at 298 K are reported in kJ mol^−1^. Calculations are at the B3LYP/6-311+G(2df,pd) (S = 6-311+G(3df)) level. Computed harmonic frequencies are scaled by a factor of 0.974, except those regarding S-X stretching modes, which are left unscaled.

The IRMPD spectrum of Zn(H_2_O+SDZ−H)^+^ is similar to that of deprotonated SDZ, as confirmed by the presence of major absorptions at comparable wavenumbers. However, given the remarkable metal-promoted ionization of water that is also known to form the basis for catalysis by the carbonic anhydrase family of enzymes (where the pKa of zinc-bound water may be as low as 6), deprotonation of the aquo ligand obviously needs to be taken into account. An extensive survey of candidate geometries converged to a most stable isomer being represented by a Zn(OH)(SDZ)^+^ complex, **OHD_1**, as shown in Figure 2. The computed IR spectrum, also depicted in Figure 2, provides a neat interpretation of the IRMPD bands. Mode assignments are listed in Table 2. Other isomers, together with their IR spectra, are also displayed in Figure 2. The **OHD_2** rotamer, differing for the hydroxyl orientation, lies 9 kJ mol^−1^ higher in free energy and does not present significant differences in the IR spectrum with respect to **OHD_1**. Isomer **OHD_3** is an imino tautomer of **OHD_1** that is less energetically favored, as typically found for N-heterocyclic arenesulfonamides [16]. All three structures are characterized by a planar three-coordinate zinc in a distorted trigonal arrangement that is coplanar with the pyrimidine ring and embedded in a rather rigid structure. SDZ behaves as a bidentate O/N(pyrimidine) ligand and presents an NH_2_ group lying on the plane of the phenyl ring. The **OH_2_D_1** isomer, lying significantly higher in energy, is a Zn(OH_2_)(SDZ−H)^+^ complex characterized by a four-coordinate zinc. Both oxygen atoms of the sulfonyl group are engaged in zinc coordination. In this complex, the two aromatic rings lie on a plane including the metal and the S atom, whereas HOH and OSO lie in a bisected fashion. On account of both the matching of the computed IR spectra and of the energy ordering of the candidate isomers, it can be concluded that the assayed ion population is best represented by **OHD_1**. Calculations at the lower B3LYP/6-311+G(d,p) level were performed on a more extensive set of isomers, including complexes where metal coordination involves the amino nitrogen. These species are all considerably higher in energy and relevant data (optimized structures, relative free energy/enthalpy and IR spectra), as reported in Figure S4a,b.

### 2.4. Structural and Vibrational Features of Zn(H_2_O+STZ−H)^+^ Complexes

**Figure 3:**
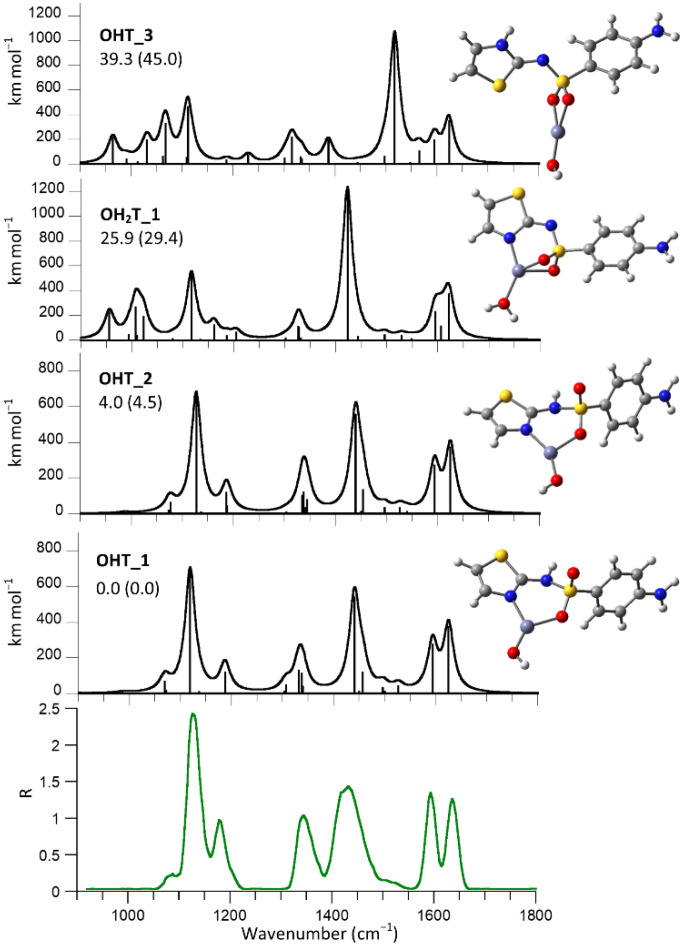
IRMPD spectrum of Zn(H_2_O+STZ−H)^+^ (bottom panel, green profile) compared with calculated IR spectra of selected isomers, the optimized structures of which are reported on the right. Relative free energies (enthalpies) at 298 K are reported in kJ mol^−1^. Calculations are at the B3LYP/6-311+G(2df,pd) (S = 6-311+G(3df)) level. Computed harmonic frequencies are scaled by a factor of 0.974, except those regarding S-X stretching modes, which are left unscaled.

Replacing SDZ with STZ introduces an asymmetry in the sulfa drug ligand due to the presence of both S and N atoms in the heterocycle, although the overall variation in structure is rather limited. Not unexpectedly, the IRMPD spectrum of Zn(H_2_O+STZ−H)^+^ presents comparable features at close wavenumbers as those already appearing in the spectrum of Zn(H_2_O+SDZ−H)^+^ (Figure 3). However, an additional band is observed at 1182 cm^−1^ that has no visible counterpart in the IRMPD spectrum of Zn(H_2_O+SDZ−H)^+^. A computational survey yielded a most stable structure conforming to a ZnOH^+^ complex with STZ (**OHT_1**), the geometry of which is strictly similar to **OHD_1**. The experimental spectrum is well interpreted by the calculated IR spectrum of **OHT_1**, which is nearly identical to the that of **OHT_2** (Figure 3). This rotamer lies slightly higher in energy, although by a reduced gap when compared to the corresponding SDZ complexes. Mode assignments listed in Table 3 show that the IRMPD band at 1182 cm^−1^ accounts for a C-S stretch in the heteroaromatic ring. No amido–imido tautomerism can occur if the heterocyclic nitrogen is coordinated to the metal. In these most stable Zn(OH)(STZ)^+^ isomers, STZ behaves as a bidentate O/N(thiazole) ligand. Replacing N(thiazole) with S(thiazole) as a chelation site yields a structure, **OHT_4**, reported in Figure S6, where data are collected on few other candidate isomers obtained at the B3LYP/6-311+G(d,p) level. **OHT_4** is considerably higher in free energy (at 91 kJ mol^−1^ relative to **OHT_1**) and displays a highly distorted trigonal zinc that is nearly perpendicular to the thiazole ring (Figure S6). Moving to the imino tautomer of **OHT_4**, geometry optimization leads to **OHT_3** (Figure 3), where the Zn-S(thiazole) distance is increased to non-bonding length, and the ensuing complex displays a three-fold O coordination at the metal. Additionally, the Zn(H_2_O+STZ−H)^+^ complex may be represented by a Zn(OH_2_)(STZ−H)^+^ isomer, **OH_2_T_1**, as depicted in Figure 3. Its geometry is characterized by 27° tilt angle between the two aromatic rings, and its relative free energy is 26 kJ mol^−1^. However, the computed IR spectrum of **OH_2_T_1** is not consistent with the experimental IRMPD spectrum, especially in the low frequency range, where significant features should be expected. The low-lying candidate isomers shown in Figure 3 clearly show that the sampled complex is well described by the thermodynamically favored isomers **OHT_1,2**.

## 3. Concluding Remarks: Ligation Motifs in SDZ- and STZ-Coordinated Zinc Complexes Assayed as Isolated Species in the Gas Phase

The most stable structure of Zn(H_2_O+SA−H)^+^ complexes, where SA = SDZ, STZ, conform to a Zn(OH)(SA)^+^ isomer in which ZnOH^+^ is coordinated to SDZ/STZ in a chelate fashion, engaging an aza group from the heterocyclic ring and a sulfonyl oxygen (**OHD_1** and **OHT_1**). This three-coordinate ligation is not a favored environment around zinc, which favors a four-coordinate tetrahedral ligation. For example, the Zn(STZ−H)_2_(H_2_O) complex displays a regular tetrahedral arrangement in the solid state, where each STZ anion chelates two zinc ions via N(thiazole) and N(amino) atoms in a bridge [51]. In the gas phase, a strain free ion, such as ZnOH^+^(H_2_O)_3_, attains a quasi-tetrahedral structure, as indicated by the IR spectrum recorded in the OH stretching range acquired using cryogenic ion infrared predissociation spectroscopy [52]. Tetrahedral zinc binding sites are also common in proteins, where zinc plays a structural role interacting with N, S and O donors from histidine, cysteine, glutamate and aspartate residues [53]. In the present study, the tetrahedral coordination of Zn(OH)(SA)^+^ complexes is impeded by the rigid structure of the SDZ/STZ ligand. A four-coordinate environment is instead attained in the Zn(OH_2_)(SA−H)^+^ isomers, **OH_2_D_1** and **OH_2_T_1**. Here the metal binding sites, besides the aquo ligand, are a nitrogen atom from the heterocycle and two oxygen atoms from the sulfonyl group. However, this ligation arrangement appears to be affected by considerable strain, with an O-Zn-O angle of 69.0° in **OH_2_D_1** and 67.8° in **OH_2_T_1**. The relative energy of the Zn(OH_2_)(SA−H)^+^ and Zn(OH)(SA)^+^ complexes may be taken as a measure of the relative acidity of the water and sulfonamide ligands in the isolated Zn(OH_2_)(SA)^2+^ complex. In the case of SDZ, the difference in free energy between **OH_2_D_1** and **OHD_1**, namely the two most stable geometries among the two isomers, amounts to 63 kJ mol^−1^, whereas the difference between **OH_2_T_1** and **OHT_1** is equal to 26 kJ mol^−1^. Thus, the acidity of SDZ and STZ, as evidenced by pK_a_ values in solution of 6.5 and 7.1, respectively, is counterbalanced (albeit to rather minor extent in terms of relative energy difference) in the gaseous Zn(OH_2_)(SA)^2+^ complex, where deprotonation of the STZ ligand is preferred relative to deprotonation of SDZ. In both cases, the acidity of water prevails, as shown by the lowest energy attached to Zn(OH)(SA)^+^ complexes. The acidity of water is well known to be strongly enhanced by complexation with Zn due to an electron pair donation to the metal. The acidity of Zn-coordinated water also emerges also in the charge separation processes undergone by isolated Zn^2+^(H_2_O)_n_ ions, leading to ZnOH^+^(H_2_O)_m_ + H^+^(H_2_O)_n−m−1_, as thoroughly explored by threshold collision-induced dissociation (CID) in a guided ion beam tandem mass spectrometer [54]. A drive towards formation of ZnOH^+^(H_2_O)_m_ also emerges in the gas phase reaction of Zn^+^(H_2_O)_n_ with acetonitrile, implying oxidation of the metal [55].

In CA enzymes, zinc promoted ionization of water is characterized by a pK_a_ in the 5.5–8 range. Thus, in a highly simplified molecular complex, characteristic properties of Zn^2+^ bound to water and prototypical sulfonamide ligands emerge and can be analyzed, yielding insight into intrinsic properties that may be masked by multifarious factors in more complex chemical and biochemical environments.

## 4. Materials and Methods

### 4.1. Sample Solutions for Electrospray Ionization

All reagents were commercial products (Sigma-Aldrich s.r.l. Milan, Italy) and were used without purification. Deprotonated sulfadiazine ions, (SDZ-H)^−^ at *m*/*z* 249, were obtained by electrospray ionization (ESI) by direct infusion of a 5 μM solution of the sulfonamide drug dissolved in water/methanol/ammonia (1:1:0.01) at a flow rate of 2 μL min^−1^. Zinc complexes, Zn(H_2_O+SDZ−H)^+^ (C_10_H_11_N_4_O_3_SZn^+^ at *m*/*z* 331-335) and Zn(H_2_O+STZ−H)^+^ (C_9_H_10_N_3_O_3_S_2_Zn at *m*/*z* 336-340), were obtained by mixing equimolar solutions (10 μM) of the selected sulfonamide and Zn(ClO_4_)_2_ in water/methanol (1:1) solvent in a 1:1 ratio.

### 4.2. IRMPD Spectroscopy

IRMPD spectroscopy of selected ions was performed using the CLIO free-electron laser (FEL) beamline. The IR radiation beamline was coupled to a hybrid FT-ICR tandem mass spectrometer (APEX-Qe Bruker) equipped with a 7.0 T actively shielded magnet, an ESI source and a quadrupole–hexapole interface [56,57]. The ions of interest were mass-selected in the quadrupole and accumulated in the hexapole-containing argon buffer gas for 0.5 s before being directed into the ICR cell. Here, irradiation of the trapped ions lasted 0.3–1s, and a mass spectrum was recorded from an accumulation over, typically, four scans. The electron energy of the FEL was set at 44.4 MeV to enhance the laser power in the selected frequency range, and a stable average power of 800–900 mW was observed. IRMPD spectra were obtained by plotting the photofragmentation yield, R (R = −ln[I_parent_/(I_parent_ + ƩI_fragment_)], where I_parent_ and I_fragment_ are the integrated abundances of the precursor and fragment ions, respectively), as a function of the wavenumber of the IR radiation.

### 4.3. Computational Methods

Tentative structures of (SDZ-H)^−^, Zn(H_2_O+SDZ−H)^+^ and Zn(H_2_O+STZ−H)^+^ were obtained by a combination of chemical intuition and conformational sampling using the conformer distribution tool in the Spartan’16 software suite [58] and the semiempirical PM6 method. Optimization of the as-obtained geometries was accomplished at the B3LYP/6-311+G(d,p) level. Selected lowest-lying isomers were subsequently reoptimized at the B3LYP level using the 6-311+G(2df,pd) basis set for all O, N, C and H atoms and the 6-311+G(3df) basis set for the S atom [29,32]. Gaussian 09 rev. D.01 was used for all density functional theory calculations [59]. Harmonic vibrational frequencies were computed at both levels of theory to obtain IR spectra and thermodynamic corrections to the electronic energies. Harmonic frequencies were scaled by 0.974 on the basis of the agreement obtained with the IRMPD spectra [27,57]. However, vibrational modes involving SX bonds were left unchanged, in agreement with evidence reported in previous works [60,61,62,63,64]. Calculated linear IR spectra were convoluted with a Lorentzian profile of 12 cm^−1^ (fwhm) to facilitate convenient comparison with the experimental IRMPD absorptions [65,66].

## Figures and Tables

**Table 1:** Observed IRMPD bands of the (SDZ−H)^−^ anion and calculated vibrational frequencies of **SDZ−H_1**.

IRMPD ^1^	Calculated SDZ−H_1 ^1,2^	Assignment
1579	1608 (101)	NH_2_ sciss
	1580 (351)	CN (pyr) stretch
1517	1505 (96)	CC (pyr) stretch
1456	1442 (1291)	C(pyr)-NSO_2_ stretch
1289	1282 (182)	SO_2_ asymm stretch
1156	1144 (347)	SO_2_ symm stretch
1103	1100 (135)	C-S stretch

^1^ In cm^−1^. ^2^ Intensities (in parentheses) in km mol^−1^.

**Table 2:** Observed IRMPD bands of the Zn(H_2_O+SDZ−H)^+^ complex and calculated vibrational frequencies of **OHD_1**.

IRMPD ^1^	Calculated OHD_1 ^1,2^	Assignment
1622	1624 (356)	NH_2_ sciss
1582	1601 (172)	C-C(pyr)+C-N(pyr) stretch
	1601 (149)	C-C(pyr)+C-N(pyr) stretch
	1594 (298)	C-C(aniline) stretch
1430	1429 (784)	C(pyr)-NSO_2_ stretch
1322	1325 (159)	SO_2_ asymm stretch
	1318 (132)	C(pyr)-NSO_2_ stretch + CH bend
1108	1111 (582)	C-S stretch + S-O stretch

^1^ In cm^−1^. ^2^ Intensities (in parentheses) in km mol^−1^.

**Table 3:** Observed IRMPD bands of the Zn(H_2_O+STZ−H)^+^ complex and calculated vibrational frequencies of **OHT_1**.

IRMPD ^1^	Calculated OHT_1 ^2^	Assignment
1637	1626 (374)	NH_2_ sciss
1595	1593 (274)	CC (aniline) stretch + NH_2_ sciss
1432	1457 (118)	NH bend + thiazole breath
	1441 (540)	NH bend
1342	1338 (112)	C-NH_2_ stretch + CH bend
	1332 (130)	SO_2_ stretch asymm + NH bend
1182	1189 (120)	C-S(thiazole) stretch + NH bend
1127	1119 (694)	C-SO_2_ stretch + S-O stretch
1087	1069 (67)	S-O stretch + C-SO_2_ stretch

^1^ In cm^−1^. ^2^ Intensities (in parentheses) in km mol^−1^.

## Data Availability

The data presented in this study are available on request from the corresponding authors.

## Supplementary Materials

The following supporting information can be downloaded at: https://www.mdpi.com/article/10.3390/molecules27103144/s1, Figure S1: Mass spectrum of the isolated deprotonated sulfadiazine ion at *m*/*z* 249 recorded (**a**) after irradiation by IR light at 1500 cm^−1^, (**b**) after irradiation at 1288 cm^−1^, and **(c)** without laser; Figure S2: Mass spectrum following isolation of Zn(H_2_O+SDZ−H)^+^ ions at *m*/*z* 331 recorded (**a**) after irradiation by IR light at 1572 cm^−1^ using an attenuator, (**b**) at the same wavelength without an attenuator and (**c**) without laser; Figure S3: Mass spectrum following isolation of Zn(H_2_O+STZ−H)^+^ ions at *m*/*z* 336 recorded (**a**) after irradiation by IR light at 1640 cm^−1^ and (**b**) without laser; Figure S4. IRMPD spectrum of (SDZ−H)^−^ (bottom panel) compared with calculated IR spectra of **SDZ−H_1** and **SDZ−H_2**, the optimized structures of which are reported on the right. Relative free energies (enthalpies) at 298 K are reported in kJ mol^−1^. Calculations are at the B3LYP/6-311+G(d,p) level; Figure S5: IRMPD spectrum of Zn(H_2_O+SDZ−H)^+^ (bottom panel) compared with calculated IR spectra of isomers, the optimized structures of which are reported on the right. Relative free energies (enthalpies) at 298 K are reported in kJ mol^−1^. Calculations are at the B3LYP/6-311+G(d,p) level; Figure S6: IRMPD spectrum of Zn(H_2_O+STZ−H)^+^ (bottom panel) compared with calculated IR spectra of isomers, the optimized structures of which are reported on the right. Relative free energies (enthalpies) at 298 K are reported in kJ mol^−1^. Calculations are at the B3LYP/6-311+G(d,p) level.
