# Supplementary material for: Ligation Motifs in Zinc-Bound Sulfonamide Drugs Assayed by IR Ion Spectroscopy
Source: Molecules. 2022 May 14;27(10):3144. doi: 10.3390/molecules27103144 (PMC9146759; doi:10.3390/molecules27103144)
Supplement: Supplementary file 1 [file molecules-27-03144-s001.zip › molecules-1694647-supplementary.pdf]

Supplementary Material for

Ligation motifs in zinc bound sulfonamide drugs assayed by IR ion spectroscopy

Davide Corinti [1*], Barbara Chiavarino [1], Philippe Maitre [2], Maria Elisa Crestoni [1], Simonetta Fornarini [1*]

[1] Dipartimento di Chimica e tecnologie del farmaco, Sapienza Università di Roma, P.le A. Moro 5, I-00185, Roma 8Italy9; davide.corinti@uniroma1.it (D.C.); barbara.chiavarino@uniroma1.it (B.C.); mariaelisa.crestoni@uniroma1.it (M.E.C.)

[2] Institut de Chimie Physique, UMR8000, CNRS, Universit8 Paris-Saclay, 91405 Orsay (France); philippe.maitre@universite-paris-saclay.fr

Figure S1. Mass spectrum of the isolated deprotonated sulfadiazine ion at *m/z* 249, recorded a) after irradiation by IR light at 1500 cm^{-1}; b) after irradiation at 1288 cm^{-1}; and c) without laser.

Figure S2. Mass spectrum following isolation of $Zn(H_2O+SDZ\text{-}H)^+$ ions at *m/z* 331, recorded a) after irradiation by IR light at 1572 cm^{-1}; b) same wavelength using an attenuator; and c) without laser.

Figure S3. Mass spectrum following isolation of $Zn(H_2O+STZ\text{-}H)^+$ ions at *m/z* 336, recorded a) after irradiation by IR light at 1640 cm^{-1}; and b) without laser.

Figure S4. IRMPD spectrum of $(SDZ\text{-}H)^-$ (bottom panel) compared with calculated IR spectra of **SDZ-H_1** and **SDZ-H_2**, whose optimized structures are reported on the right. Relative free energies (enthalpies) at 298 K are reported in kJ mol^{-1}. Calculations are at the B3LYP/6-311+G(d,p) level.

Figure S5a-b. IRMPD spectrum of $Zn(H_2O+SDZ\text{-}H)^+$ (bottom panel) compared with calculated IR spectra of isomers whose optimized structures are reported on the right. Relative free energies (enthalpies) at 298 K are reported in kJ mol^{-1}. Calculations are at the B3LYP/6-311+G(d,p) level.

Figure S6. IRMPD spectrum of $Zn(H_2O+STZ\text{-}H)^+$ (bottom panel) compared with calculated IR spectra of isomers whose optimized structures are reported on the right. Relative free energies (enthalpies) at 298 K are reported in kJ mol^{-1}. Calculations are at the B3LYP/6-311+G(d,p) level.

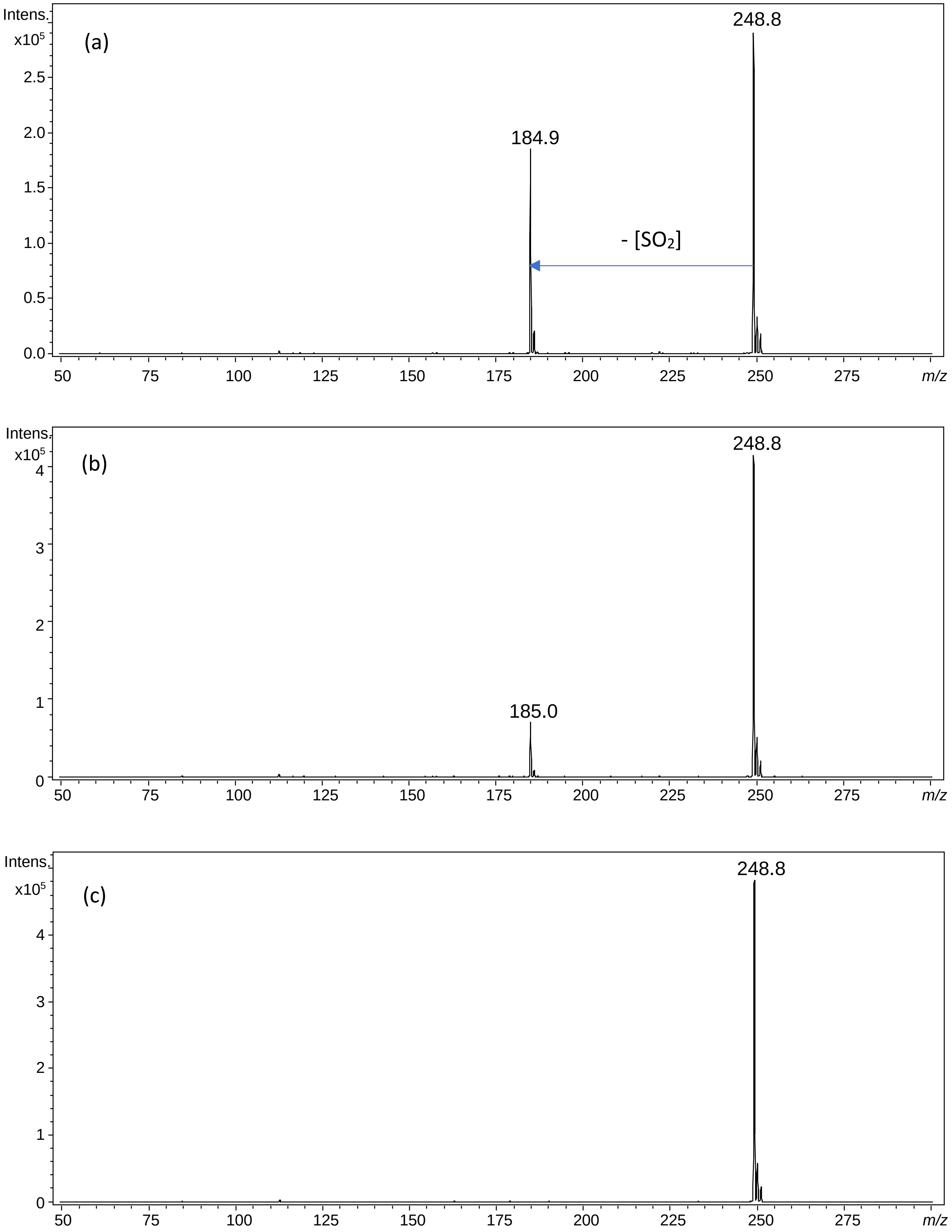

Figure S1. Mass spectrum of the isolated deprotonated sulfadiazine ion at *m/z* 249, recorded a) after irradiation by IR light at 1500 cm^{-1}; b) after irradiation at 1288 cm^{-1}; and c) without laser.

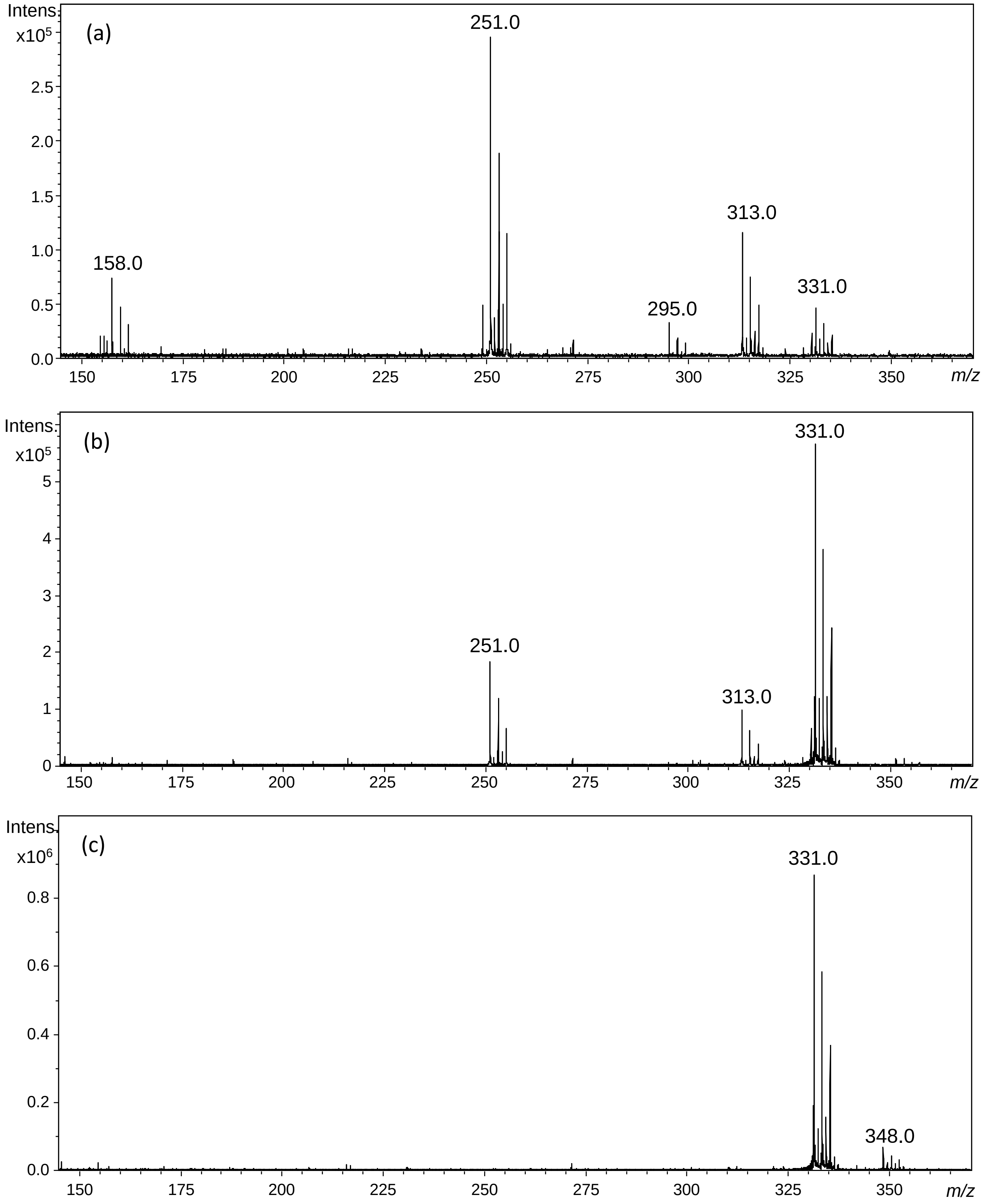

Figure S2. Mass spectrum following isolation of Zn(H_2O+SDZ-H)$^+$ ions at *m/z* 331, recorded a) after irradiation by IR light at 1572 cm^{-1}; b) same wavelength using an attenuator; and c) without laser. The full isotopic cluster is selected and submitted to IR laser radiation.

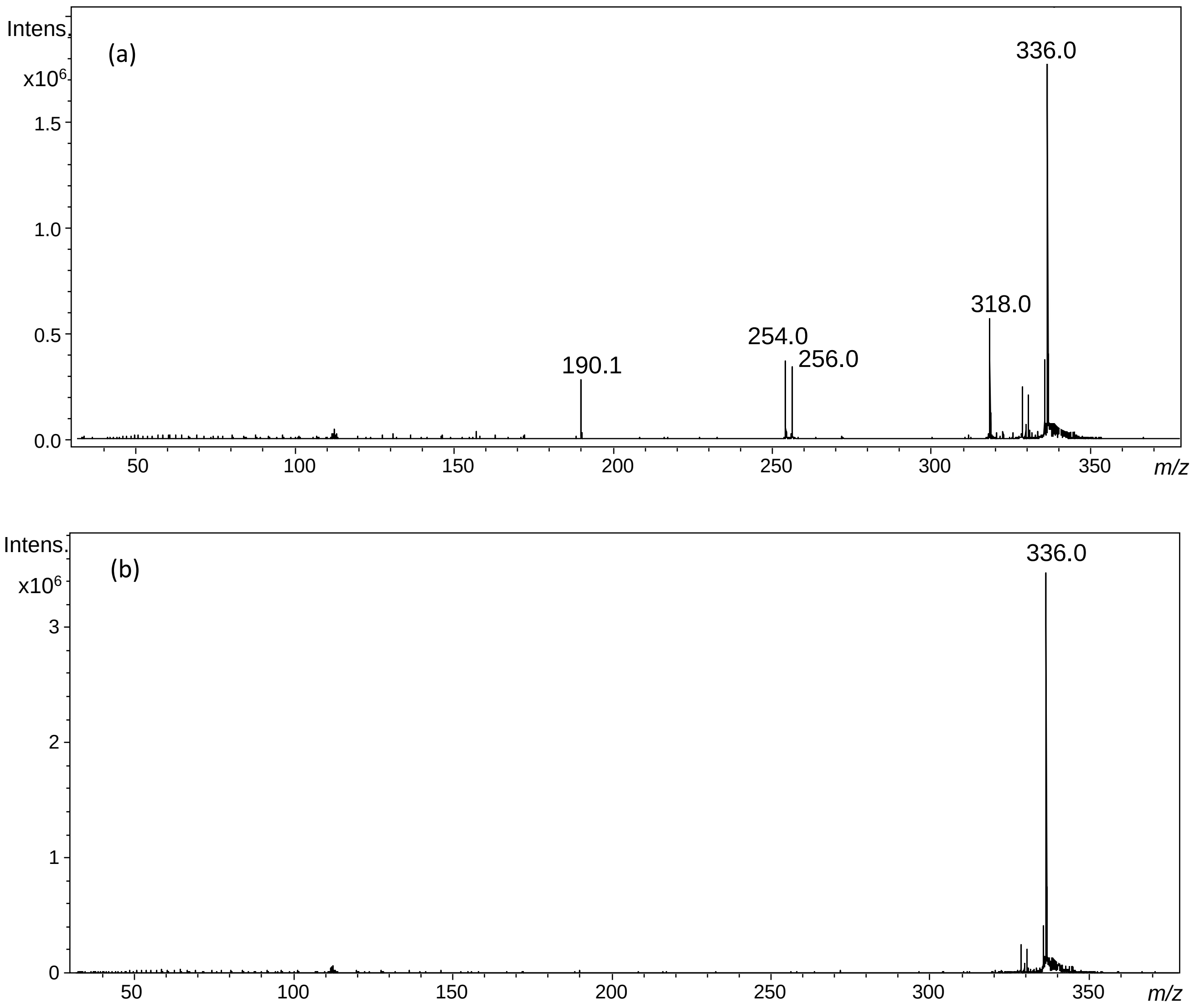

Figure S3. Mass spectrum following isolation of Zn(H_2O+STZ-H)$^+$ ions at *m/z* 336, recorded a) after irradiation by IR light at 1640 cm^{-1}; and b) without laser. The monoisotopic ion ($^{12}C_9H_{10}{}^{14}N_3{}^{16}O_3{}^{32}S_2{}^{64}Zn$)$^+$ at *m/z* 336 is selected and submitted to IR laser radiation.

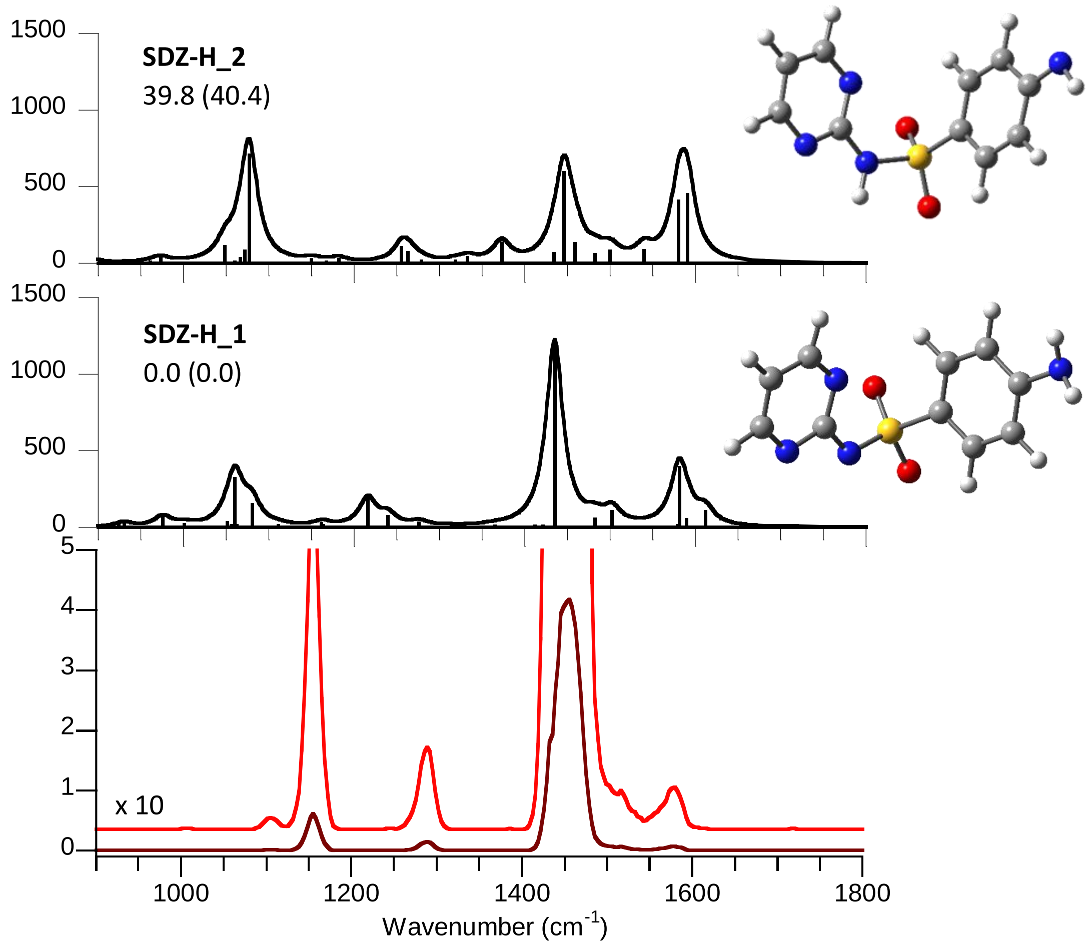

Figure S4. IRMPD spectrum of (SDZ-H)$^{-}$ (bottom panel) compared with calculated IR spectra of **SDZ-H_1** and **SDZ-H_2**, whose optimized structures are reported on the right. Relative free energies (enthalpies) at 298 K are reported in kJ mol^{-1}. Calculations are at the B3LYP/6-311+G(d,p) level. Computed harmonic frequencies are scaled by a factor of 0.974 except those regarding S-X stretching modes which are left unscaled.

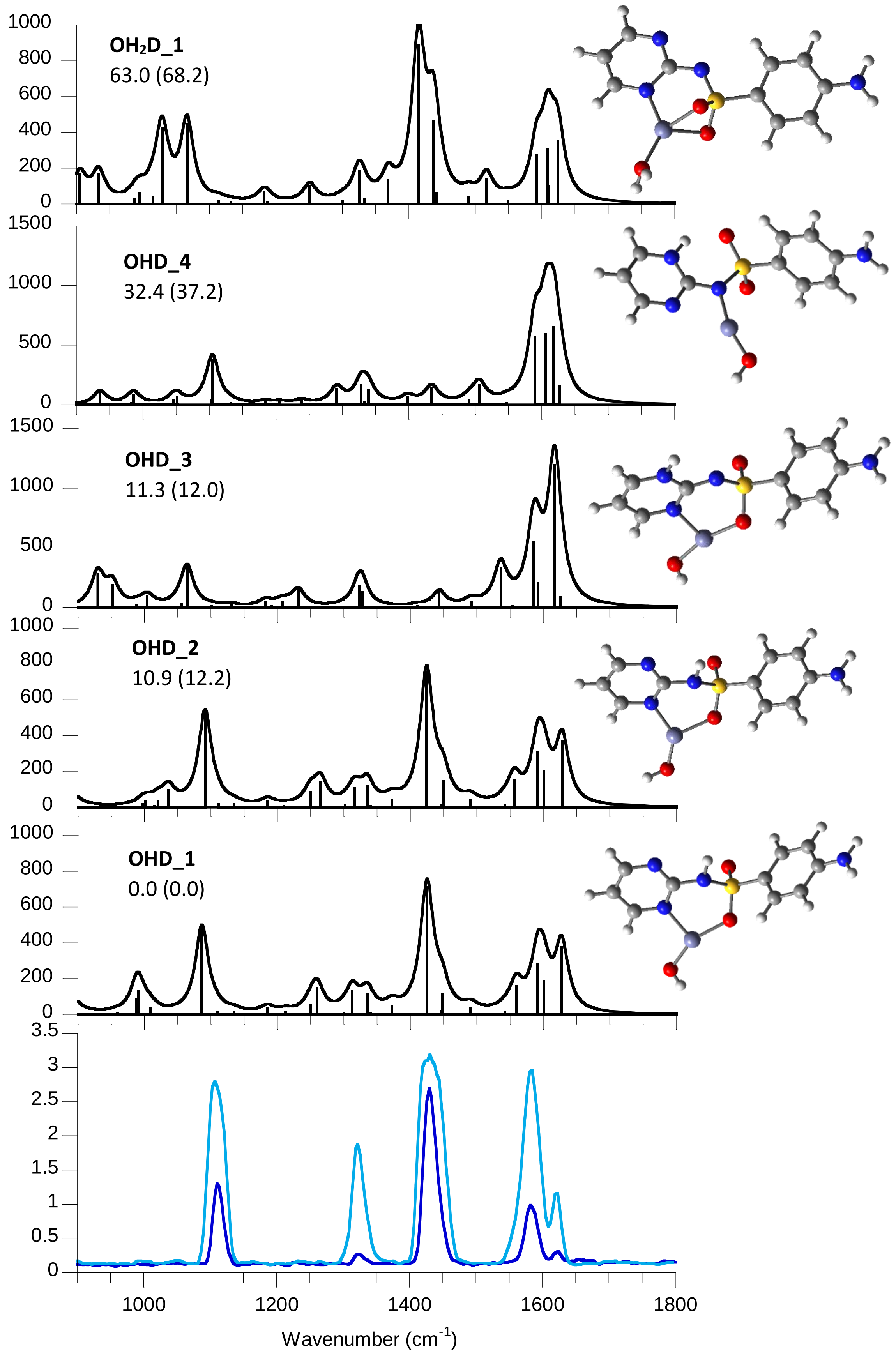

Figure S5a. IRMPD spectrum of Zn(H_2O+SDZ-H)$^+$ (bottom panel) compared with calculated IR spectra of isomers whose optimized structures are reported on the right. Relative free energies (enthalpies) at 298 K are reported in kJ mol^{-1}. Calculations are at the B3LYP/6-311+G(d,p) level. Computed harmonic frequencies are scaled by a factor of 0.974 except those regarding S-X stretching modes which are left unscaled.

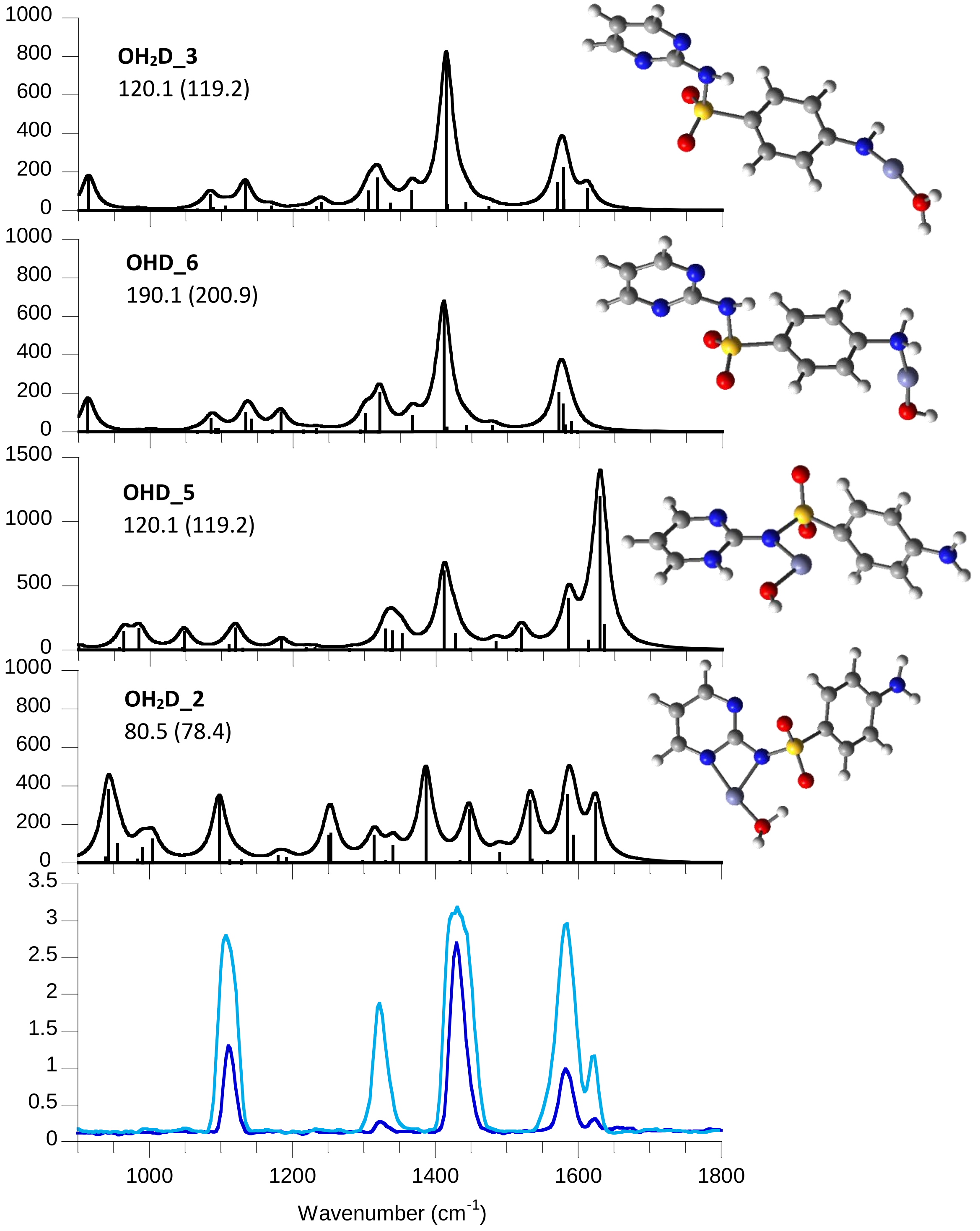

Figure S5b. IRMPD spectrum of $Zn(H_2O/SDZ\text{-}H)^+$ (bottom panel) compared with calculated IR spectra of isomers whose optimized structures are reported on the right. Relative free energies (enthalpies) at 298 K are reported in kJ mol^{-1}. Calculations are at the B3LYP/6-311+G(d,p) level. Computed harmonic frequencies are scaled by a factor of 0.974 except those regarding S-X stretching modes which are left unscaled.

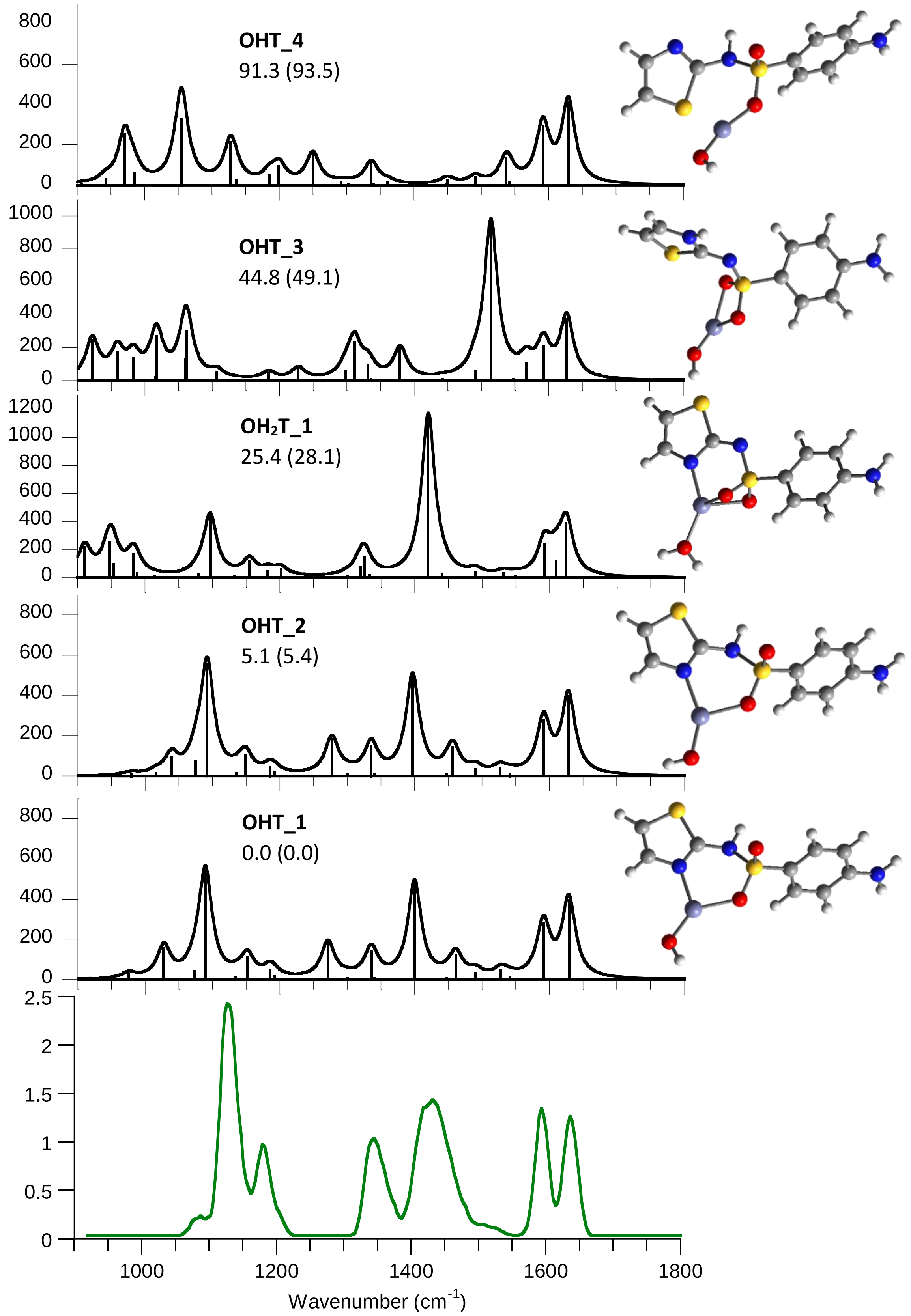

Figure S6. IRMPD spectrum of $Zn(H_2O+STZ-H)^+$ (bottom panel) compared with calculated IR spectra of isomers whose optimized structures are reported on the right. Relative free energies (enthalpies) at 298 K are reported in kJ mol^{-1}. Calculations are at the B3LYP/6-311+G(d,p) level. Computed harmonic frequencies are scaled by a factor of 0.974 except those regarding S-X stretching modes which are left unscaled.
